# Dietary Acrylamide Intake and the Risk of Pancreatic Cancer: The Japan Public Health Center-Based Prospective Study

**DOI:** 10.3390/nu12113584

**Published:** 2020-11-22

**Authors:** Kumiko Kito, Junko Ishihara, Ayaka Kotemori, Ling Zha, Rong Liu, Norie Sawada, Motoki Iwasaki, Tomotaka Sobue, Shoichiro Tsugane

**Affiliations:** 1 Graduate School of Environmental Health, Azabu University, 1-17-71 Fuchinobe, Chuo-ku, Sagamihara, Kanagawa 252-5201, Japan; de1801@azabu-u.ac.jp; 2 Department of Food and Life Science, Azabu University, 1-17-71 Fuchinobe, Chuo-ku, Sagamihara, Kanagawa 252-5201, Japan; kotemori@azabu-u.ac.jp; 3 Department of Environmental Medicine and Population Sciences, Graduate School of Medicine, Osaka University, 2-2 Yamadaoka, Suita, Osaka 565-0871, Japan; sarin@envi.med.osaka-u.ac.jp (L.Z.); liur8939@163.com (R.L.); tsobue@envi.med.osaka-u.ac.jp (T.S.); 4 Epidemiology and Prevention Group, Center for Public Health Sciences, National Cancer Center, 5-1-1 Tsukiji, Chuo-ku, Tokyo 104-0045, Japan; nsawada@ncc.go.jp (N.S.); moiwasak@ncc.go.jp (M.I.); stsugane@ncc.go.jp (S.T.)

**Keywords:** acrylamide, Asia, pancreatic cancer, diet, epidemiologic study

## Abstract

Acrylamide is a probable carcinogen in humans. Few studies have assessed dietary acrylamide intake and the risk of pancreatic cancer; however, these studies are based on Western populations. Our purpose was to investigate the association of dietary acrylamide intake with the risk of pancreatic cancer utilizing data from the Japan Public Health Center-based Prospective Study. We evaluated the data of 89,729 participants aged 45–74 years, who replied to a questionnaire on past medical history and lifestyle habits from 1995–1998. Dietary acrylamide intake was estimated utilizing a validated food frequency questionnaire. We calculated the hazard ratios and 95% confidence intervals by using Cox proportional-hazards regression models. The average follow-up was 15.2 years, and 576 cases of pancreatic cancer were diagnosed. In the multivariate-adjusted model, an association between dietary acrylamide intake and pancreatic cancer risk was not demonstrated (hazard ratio for the highest vs. lowest quartile = 0.83, 95% confidence interval: 0.65–1.05, *p* for trend = 0.07). Furthermore, in the analyses stratified by sex, smoking status, coffee consumption, green tea consumption, alcohol consumption, and body mass index, no significant association was detected. Dietary acrylamide intake was not associated with the pancreatic cancer risk in Japanese individuals.

## 1. Introduction

In 1994, acrylamide was classified by the International Agency for Research on Cancer Group as a 2A agent, which means that it is probably carcinogenic in humans [1]. The leading causes of acrylamide exposure are regarded to be tobacco smoking and acrylamide-containing foods [2,3]. Acrylamide in foods is mainly formed during the Maillard reaction, in which asparagine (an amino acid) reacts with reducing sugars such as glucose in the presence of heat (120 °C or more) [2]. Acrylamide is partly metabolized to glycidamide by cytochrome P450 (CYP2E1). Glycidamide is known to cause genotoxicity by forming an adduct that binds to hemoglobin and DNA [4,5].

Currently, there are a few studies that have investigated the association between pancreatic cancer risk and dietary acrylamide intake. Five studies (two prospective cohort studies, one case-cohort study, one case-control study, and one pooled analysis of six case-control studies) have assessed the association of acrylamide intake with pancreatic cancer [6,7,8,9,10]; however, no such association has been established. This finding is supported by a recent meta-analysis, which showed that dietary acrylamide intake was not associated with pancreatic cancer risk [11]. However, in each of these studies, stratification by obesity status yielded inconsistent results. For an increment of 10 μg/d of acrylamide, one study reported a negative association with the pancreatic cancer risk [7], another reported a raised risk [8], and two studies observed no association [9,10]. Studies on the association of pancreatic cancer risk with dietary acrylamide, including the stratified analyses, are limited; therefore, more studies are needed to support these results.

All the prior studies were performed in Western countries and the association between dietary acrylamide and the risk of pancreatic cancer among Asians, has not been examined. Acrylamide intake and the food groups that contribute to acrylamide intake are different in the Japanese population compared with the Western population [12]. Japanese people have an acrylamide intake of 7–10 μg/d [12], which is less than half of that of westerners [6,7,8,10]. In Japan, food groups that mainly contribute to acrylamide intake are green tea, coffee, confectionery, vegetables, and potatoes [12], whereas in Western countries they are potatoes, bread, cakes, and coffee [13,14]. Considering its potential carcinogenicity, there is a need to evaluate the association of dietary acrylamide intake with pancreatic cancer by targeting populations with different dietary sources and acrylamide intakes.

The present study aimed to identify the association between dietary acrylamide intake and pancreatic cancer in Japanese individuals.

## 2. Materials and Methods

### 2.1. Study Participants

The Japan Public Health Center-based Prospective Study (JPHC study) was initiated in the 1990s. It was a large population-based cohort study that aimed to explore the association between lifestyle habits and lifestyle-related diseases. The JPHC study had two cohorts covering 11 public center areas. Cohort Ⅰ covered areas including Iwate, Akita, Nagano, Okinawa-Chubu, and Tokyo. Cohort II covered areas including Ibaraki, Niigata, Kochi, Nagasaki, Okinawa-Miyako, and Osaka. At baseline, the participants were 140,420 residents (68,722 men and 71,698 women) aged 40–69 years. A self-administered food frequency questionnaire (FFQ) was conducted at the dietary survey at the baseline and at 5 and 10 years of the follow-up. We used the FFQ of the 5-year follow-up survey as the starting point because it provided information about more food items and portion size options than the FFQ of the baseline. The study protocol has been documented in detail elsewhere [15,16]. The protocol of the present study was approved by the institutional review boards of the National Cancer Center, Japan (ethical approval number: 2001-021), Osaka University (approval number: 14020-9), and Azabu University (approval number: 2527). All study participants provided informed consent before participating in the study.

**Figure 1:**
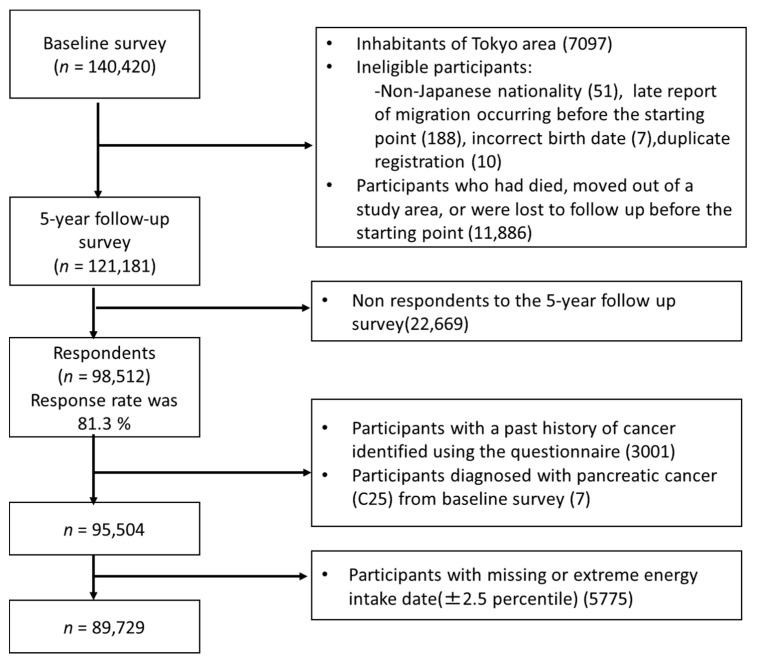
Flow diagram of the study participant selection.

In this study, we excluded inhabitants from the Tokyo area because cancer incidence data were unavailable. We also excluded participants who were of non-Japanese nationality, had a late report of migration before the starting point, an incorrect birth date, died, moved out of the study area, or were lost to follow-up before the starting point. In the present study, 121,181 participants were eligible for inclusion in the analysis. Of those, 98,512 responded to the 5-year follow-up survey (response rate, 81.3%). Furthermore, we excluded the following participants; those with a history of cancer (*n* = 3001), with pancreatic cancer (*n* = 7) before the 5-year follow-up survey, and with missing or extreme energy intake data (*n* = 5775). The final analysis included 89,729 (men: 42,071, women: 47,658) participants (Figure 1).

### 2.2. Food Frequency Questionnaire

We used the FFQ consisting of 138 food and beverage items to calculate the energy and dietary acrylamide intake. The participants were asked about their frequencies of eating and portion sizes during the previous year. The frequencies of eating were categorized into nine categories (never, 1–3 times/month, 1–2 times/week, 3–4 times/week, 5–6 times/week, once/day, 2–3 times/day, 4–6 times/day, or ≥7 times/day), and the portion sizes were categorized into three categories (less than half the standard portion size, standard portion size, or >1.5 times the standard portion size). Each beverage was categorized into nine categories of frequency (<1 cup/week, 1–2 cups/week, 3–4 cups/week, 5–6 cups/week, 1 cup/day, 1–3 cups/day, 4–6 cups/day, 7–9 cups/day, or ≥10 cups/day).

The intake for each food and beverage was calculated by the multiplication of the eating frequency and portion size.

### 2.3. Assessment of Energy and Acrylamide Intake from the FFQ

The energy content in each food item was based on the Fifth Revised and Enlarged Edition of the Standard Tables of Food Composition in Japan [17]. The validity of the FFQ for energy intake using the 28-day dietary records was as follows: 0.53 for men and 0.41 for women in Cohort I (*n* = 113), and 0.36 for men and 0.24 for women in Cohort II (*n* = 176) [18,19,20].

Acrylamide intake for individuals was estimated utilizing the database derived from the published acrylamide measurements in common Japanese foods [12]. In the FFQ, the following food and beverage items contained dietary acrylamide: miso, beer, baked fish paste, bread, rice cake, Japanese-style confectionery, cake, biscuits/cookies, chocolate, peanuts, fried tofu, green tea, oolong tea, black tea, coffee, and soup [21,22,23,24,25,26,27,28,29]. To further estimate the accuracy of measurement of the acrylamide intake, we estimated the acrylamide intake of each participant considering the acrylamide intake generated from homemade cooking for the following foods: heated starchy vegetables (potato and sweet potato), vegetables (onion, bean sprouts, sweet pepper, squash, cabbage, snap beans, and broccoli), toast, boiled or stir-fried rice, and fried batter [12]. The Spearman rank correlation coefficients for energy-adjusted acrylamide intake between dietary records and FFQ ranged from 0.34–0.48 for validity and 0.56–0.62 for reproducibility. Details of the database have been previously documented elsewhere [12].

### 2.4. Follow-Up and Identification of Pancreatic Cancer

The study participants were followed from the starting point of the 5-year follow-up survey (1995 for Cohort I and 1998 for Cohort II) until 31 December 2013 (until 31 December 2012, only in the Osaka area). Residential status was ascertained annually by the residential registry. During the follow-up period, 16,030 participants (17.9%) died, 5 694 (6.4%) moved from the study area, and 74 (0.1%) were lost to follow-up.

Cases of pancreatic cancer were determined through active patient information from major local hospitals in each study area and data linkage with population-based cancer registries. Death certificates were used as sources of additional information. We defined pancreatic cancer cases based on the International Classification of Diseases for Oncology, Third Edition codes C25.0–C25.9, but excluded those with C25.4 (endocrine tumor) because of its different etiology. The proportion of cases determined using Death Certificate Only was 11.5%. During a mean follow-up of 15.2 years, 576 pancreatic cancer cases were identified.

### 2.5. Statistical Analyses

We determined the person-years of follow-up for each participant from the date of the starting point to the date of diagnosis of pancreatic cancer, death, relocation from the study area, or the end follow-up (31 December 2012 for the Osaka area and 31 December 2013 for all other areas), whichever came first.

We estimated hazard ratios (HRs) and 95% confidence intervals (CIs) using the Cox proportional hazards model for energy-adjusted acrylamide intake and pancreatic cancer risk by quartile. The lowest group was used as the reference for each group. Trends in the HRs were estimated by allocating ordinal scores to the quartiles of acrylamide intake. Acrylamide intake was adjusted for total energy intake using the residual method [30]. Utilizing the known risk factors or potential confounding factors of pancreatic cancer, the model was adjusted for the following variables: age (continuous), sex, study area (10 public health center areas), smoking status (never, past, current <20, 20–39, and ≥40 cigarettes/day, or missing), alcohol intake (0, <150 g/week or ≥150 g/week, or missing), body mass index (BMI; <25 kg/m^2^, ≥25 kg/m^2^, or missing), family history of pancreatic cancer (yes or no), and medical history of diabetes mellitus (yes or no). These variables were derived from the FFQ. Red meat consumption, processed meat consumption, and physical activity have also been proposed as potential risk factors for pancreatic cancer [31], although we did not include them in the model because their inclusion did not change the HR by at least 10%. For the sensitivity analysis in the multivariate-adjusted model, we conducted the same analysis by excluding cases diagnosed during the first 3 years of follow-up. We also performed subgroup analyses to confirm the effect of interaction based on sex, smoking status (current or past smokers, never smokers), coffee consumption (<1 cup/week, ≥1 cup/week), green tea consumption (<1 cup/week, ≥1 cup/week), alcohol consumption (<150 g/week, ≥150 g/week), and BMI (<25 kg/m^2^, ≥25 kg/m^2^). Statistical analyses were conducted using SAS version 9.4 (SAS Institute Inc., Cary, NC, USA). All *p*-values were two-sided, and the statistical significance level was set at *p* < 0.05.

## 3. Results

Table 1 shows the participant characteristics by quartile for total acrylamide intake. The overall median acrylamide intake was 6.12 μg/d (IQR, 4.30–8.72), and the mean (SD) was 6.92 ± 3.81 μg/d, corresponding to 0.13 ± 0.16 μg/kg body weight/day. The group with the highest acrylamide intake (Q4) was younger, had a higher proportion of current smokers, and had a lower proportion of individuals with a history of diabetes. Regarding dietary intake, the Q4 group had a lower intake of alcohol and a higher intake of coffee, green tea, biscuits/cookies, and potatoes.

**Figure 2:**
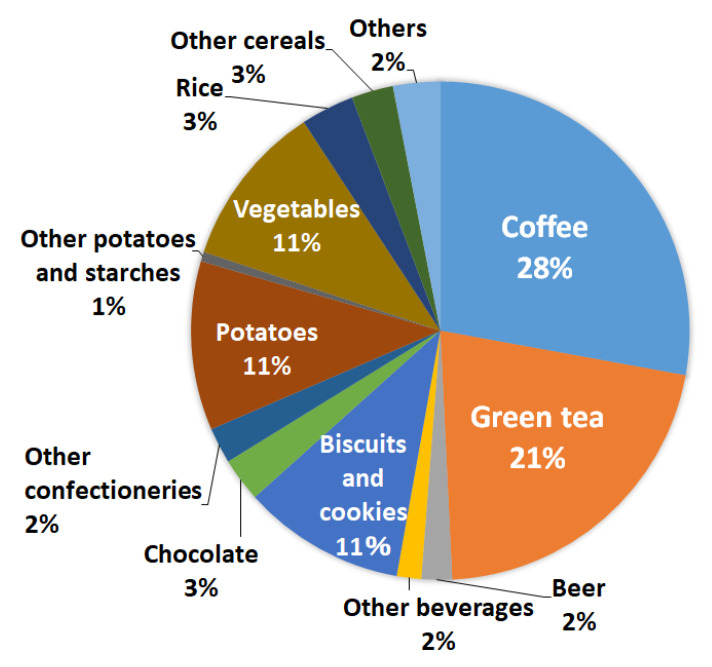
Percentage contribution of acrylamide-containing foods to dietary acrylamide intake.

**Figure 3:**
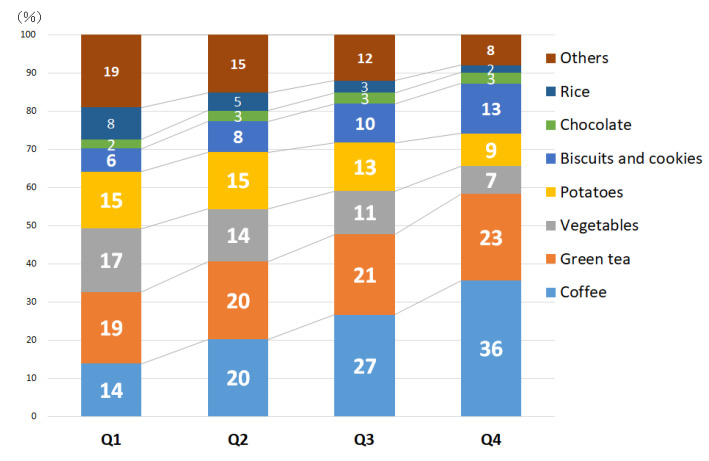
Comparison of the percentage contribution of acrylamide-containing foods to dietary acrylamide intake among quartiles of acrylamide intake.

Figure 2 and Figure 3 show the proportion contribution of foods to dietary acrylamide intake in the entire study participant and each quartile. Overall, the food groups that contributed the most to acrylamide intake were beverages (total 53%; 28% for coffee, 21% for green tea, 2% for beer, and 2% for others), followed by confections (total 16%; 11% for biscuits/cookies, 3% for chocolate, and 2% for others), vegetables (total 11%; 3% for sweet pepper, 3% for onion, 3% for bean sprouts, and 2% for others), and potatoes (11%). In each acrylamide intake group, green tea was the main contributing food, although the trends of the other foods differed slightly. The proportion of coffee and biscuits or/and cookies increased linearly (coffee: Q1: 14%, Q4: 36%; biscuits or/and cookies: Q1: 6%, Q4: 13%), whereas the proportion of vegetables decreased linearly (vegetables: Q1: 17%, Q4: 7%).

We observed no association between dietary acrylamide intake and pancreatic cancer (Table 2). In the multivariate-adjusted model, the HR of the highest quartile vs. the lowest was 0.83 (95% CI: 0.65–1.05) (*p* for trend = 0.07). This risk did not alter even after excluding cancer diagnosed cases within the first 3 years of follow-up. We also conducted stratified analyses using the major confounding factors; however, there were no significant associations in the multivariate-adjusted model: current or past smoking status (*p* for trend = 0.25), never smoking status (*p* for trend = 0.23), consumption of <1 cup/week of coffee (*p* for trend = 0.11), consumption of ≥1 cup/week of coffee (*p* for trend = 0.15), consumption of <1 cup/week of green tea (*p* for trend = 0.23), consumption of ≥1 cup/week of green tea (*p* for trend = 0.18), consumption of <150 g/week of alcohol (*p* for trend = 0.20), consumption of ≥150 g/week of alcohol (*p* for trend = 0.19), BMI <25 kg/m^2^ (*p* for trend = 0.11), and BMI ≥25 kg/m^2^ (*p* for trend = 0.71). We performed a sensitivity analysis since the proportion of coffee and biscuits/cookie to dietary acrylamide increased from Q1 to Q4. We added coffee consumption (continuous) and biscuits/cookies intake (continuous) to the multivariate-adjusted model, and in the coffee stratification analysis, we added biscuits/cookies intake (continuous). The results did not change (data not shown).

## 4. Discussion

We analyzed the association between dietary acrylamide intake and the risk of pancreatic cancer using the FFQ in a large-scale, population-based study of Japanese individuals. The analyses showed that acrylamide intake was not associated with the risk of pancreatic cancer. We also observed no association in the analyses stratified based on the smoking status, coffee intake, green tea intake, alcohol consumption, and BMI.

In the present study, the mean daily intake of acrylamide was 6.92 ± 3.81 μg/d (Q1 = 3.1± 0.9 μg/d, Q2 = 5.2± 0.5 μg/d, Q3 = 7.3 ± 0.7 μg/d, and Q4 = 12.1 ± 3.5 μg/d), which is considerably lower than that reported in studies from Western countries (26.22 ± 14.79 μg/d in the European Prospective Investigation into Cancer and Nutrition [EPIC] cohort study [7] and 22.1 ± 12.7 μg/d in the Netherlands Cohort Study on diet and cancer [NLCS] [8]). However, the mean acrylamide intake in the Q4 (highest) group of this study corresponds to approximately 25% tile of acrylamide intake in the Western population [10]. Previous studies that assessed dietary acrylamide intake and cancer risk (such as breast cancer and digestive system cancer) in the Japanese population have consistently indicated a null association [32,33,34]. Our results supported the findings of the previous studies on acrylamide intake and cancer risk in the Japanese population.

To date, only five studies on the association of acrylamide intake with pancreatic cancer using an FFQ have been undertaken in Western countries. No association has been observed in all these studies including the recent ones: a large-scale prospective (EPIC) cohort (865 pancreatic cancer cases, HR: 0.77, 95% CI: 0.58–1.04) [7], a recent meta-analysis (1732 pancreatic cancer cases, HR: 0.93, 95% CI: 0.76–1.12) [11], and a pooled analysis of six case-control studies (1975 pancreatic cancer cases, odds ratio: 0.92, 95% CI: 0.66–1.28) [10]. The population of the present study differs from those of the previous studies with respect to the characteristics of acrylamide intake. First, the mean acrylamide intake was lower. Second, the main foods contributing to total acrylamide intake included green tea and vegetables, which differed from the findings of studies conducted in Western countries. Furthermore, in Japan, vegetables are often cooked using methods such as stir-frying, baking, and frying, which can contribute to acrylamide intake [12]. However, as in previous studies, a consistent trend was observed in our study. Therefore, there might not be a substantial difference in susceptibility to acrylamide intake between Asian and Western populations.

In the analyses stratified by BMI (<25 kg/m^2^ and ≥25 kg/m^2^), we found no association of acrylamide intake with pancreatic cancer. CYP2E1, which is involved in the metabolism of acrylamide to glycidamide, has been reported to be activated at high levels of BMI [35,36]. Previous analyses of acrylamide intake associations with pancreatic cancer risk, stratified by BMI have yielded conflicting results. The EPIC study showed an inverse association between pancreatic cancer risk (lowest vs. highest HR = 0.32, 95% CI: 0.16–0.63) [7], while the PanC4 study (a pooled analysis of six case-control studies conducted in Europe, the United States, and Australia) showed no association (lowest vs. highest HR = 0.90, 95% CI: 0.46–1.78) [10]. Additionally, in the NLCS, an effect modification by obesity was observed (HR for each 10 μg/d increment: 1.59, 95% CI: 0.87–2.89, *p* for effect modification = 0.04) [8]. This study found no association of dietary acrylamide intake with pancreatic cancer in the analysis stratified by BMI.

In addition, we conducted analyses stratified by alcohol consumption; however, no association was confirmed. Alcohol reportedly inhibits CYP2E1 activity, probably competing with acrylamide as a substrate for acrylamide metabolism [37]. In fact, it has been suggested that people who consume more alcohol tend to have a lower HbGA/HbAA ratio [38,39,40]. In the analyses stratified by alcohol consumption in previous studies (the Italian case-control study [9] and EPIC study [7]), no association and some interactions with alcohol intake were suggested, respectively. Collectively, although the results of the stratification analyses of BMI and alcohol consumption are not completely consistent, further studies are needed on the possibility of confounding and modification of the effect. This is because these factors are possible risk factors of pancreatic cancer [31] and may also influence acrylamide metabolism [38,39,40].

Furthermore, as shown in Figure 2 and Figure 3, coffee and green tea ranked first and second, respectively, among the foods that contribute to total acrylamide intake. Particularly, in the Q4 (highest) group, the proportion of total acrylamide intake was 36% for coffee and 23% for green tea. Green tea is a characteristic contributory food in Japan, and coffee is a major contributory food in Western countries. Tea polyphenols have been reported to reduce HbAA concentrations in animals [41], and the EPIC study also indicated an inverse correlation between tea intake and HbAA and HbGA concentrations [38]. We observed no association of dietary acrylamide intake with pancreatic cancer in our analyses stratified by coffee and green tea consumption.

The strengths of this study are attributed to some features of the JPHC Study and are as follows: this was a prospective cohort study; the participants were recruited from a general population; it had a long follow-up period; and the response rate was high (81.3%). In addition, there was no recall bias for exposure because the data were gathered before the diagnosis of cancer.

The present study had several limitations. First, there is a possibility of measurement error resulting from the exposure assessment of acrylamide intake by FFQ. We could not take into account dietary changes of the participants during the follow-up because acrylamide intake was assessed only once. However, we inferred that their dietary habits did not change much because the dietary habits of the study participants (aged 40 years and older) are likely to be well established. In addition, the FFQ was not designed to consider cooking temperature or cooking time, which can affect acrylamide concentration in foods [42]; Cooked vegetables contribute to acrylamide intake in the Japanese population [12]. While we could not include all vegetables in this study, acrylamide intake was estimated for some foods by considering cooking methods such as baking and frying [12]. Moreover, although the present study was conducted in the 1990s, the estimation of dietary acrylamide intake utilizing the FFQ was based on a database of measurements obtained from the 2000s. Second, the number of pancreatic cancer cases may not have been adequate to perform an analysis stratified by subgroups. Accordingly, these results may have been affected by the statistical power, and the results should be interpreted with caution. Finally, the results may have been affected by unmeasured confounders. We examined the analytical model including the major risk factors (such as red meat consumption, saturated fatty acid consumption, and physical activity) so far reported [31], although they were unrelated (data not shown). However, studies using biomarkers have identified factors that influence acrylamide metabolism, such as BMI levels and alcohol intake [38,39,40,43]. Therefore, multiple factors related to diet and lifestyle may affect acrylamide metabolism, and further studies are required to explore factors that affect acrylamide metabolism using biomarkers.

## 5. Conclusions

In a large Japanese cohort study, no association was found between pancreatic cancer risk and dietary acrylamide intake, regardless of sex, smoking status, coffee intake, green tea intake, alcohol consumption, and BMI. Our results indicate that dietary acrylamide intake is not likely to increase the risk of pancreatic cancer in the Japanese population, which has a relatively lower dietary acrylamide intake than the Western population.

## Figures and Tables

**Table 1:** Baseline characteristics of the study participants (*n* = 89,729) according to quartile of dietary acrylamide intake.

	Quartile of Acrylamide Intake																			
	Quartile 1					Quartile 2					Quartile 3					Quartile 4				
Participants, *n*	22,432					22,432					22,433					22,432				
Male, %	27.8					24.5					23.3					24.3				
Female, %	22.5					25.4					26.5					25.6				
Acrylamide intake																				
Mean, µg/day ^a^	3.1	±	0.9			5.2	±	0.5			7.3	±	0.7			12.1	±	3.5		
Median, µg/day ^b^	3.3		(2.6	–	3.8)	5.2		(4.7	–	5.6)	7.2		(6.6	–	7.9)	11.0		(9.7	–	13.3)
Mean, µg/kg body weight/day ^a^	0.06	±	0.04			0.10	±	0.09			0.13	±	0.09			0.22	±	0.26		
Age at 5-year follow-up survey, years ^b^	58		(52	–	63)	57		(51	–	63)	56		(50	–	62)	55		(48	–	61)
Body mass index, kg/m^2 b^	23.4		(21.6	–	25.5)	23.4		(21.5	–	25.4)	23.3		(21.4	–	25.3)	23.1		(21.3	–	25.2)
Smoking status, %																				
Never smoker	60.6					64.5					64.4					58.9				
Ex-smoker	9.6					9.1					8.0					8.0				
Current smoker	23.2					20.6					21.9					27.7				
Missing	6.6					5.8					5.7					5.5				
History of diabetes mellitus, %	8.2					7.1					6.3					5.6				
Family history of pancreatic cancer, %	0.3					0.4					0.4					0.3				
Dietary intake																				
Energy, kcal/d ^a,c^	1999	±	642			1998	±	610			2013	±	612			1977	±	622		
Alcohol intake, g/week ^a^	155	±	250			108	±	197			90	±	175			70	±	146		
Coffee, g/d ^a,c^	34	±	50			81	±	82			144	±	131			324	±	313		
Green tea, g/d ^a,c^	285	±	314			460	±	407			559	±	449			802	±	732		
Potato, g/d ^a,c^	9	±	9			16	±	13			19	±	17			21	±	25		
Biscuits and cookies, g/d ^a,c^	1	±	1			2	±	2			3	±	3			5	±	8		
Vegetables, g/d ^a,c^	178	±	118			208	±	119			221	±	128			221	±	138		

Abbreviation: SD, standard deviation. ^a^ Data are presented as the mean (standard deviation). ^b^ Data are presented as the median (interquartile range). ^c^ Energy adjusted intake by residual method.

**Table 2:** Hazard ratios and 95% confidence intervals for pancreatic cancer risk according to quartile of acrylamide intake.

		Quartile 1		Quartile 2		Quartile 3		Quartile 4		*p* for Trend
	Total	HR	(95% CI)	HR	(95% CI)	HR	(95% CI)	HR	(95% CI)	
All										
Participants, *n*	89,729	22,432		22,432		22,433		22,432		
Cases, *n*	576	180		143		126		127		
Person-years	1,360,237	340,654		341,672		340,712		337,198		
Age- and area-adjusted ^a^		1.00	(Reference)	0.83	(0.66–1.03)	0.77	(0.61–0.97)	0.84	(0.67–1.07)	0.09
Multivariate-adjusted ^b^		1.00	(Reference)	0.84	(0.67–1.05)	0.77	(0.61–0.97)	0.83	(0.65–1.05)	0.07
Multivariate-adjusted (excluding cases <3 y) ^b^		1.00	(Reference)	0.83	(0.66–1.06)	0.77	(0.60–0.99)	0.82	(0.63–1.05)	0.08
Male										
Cases, *n*	319	108		70		67		74		
Multivariate-adjusted ^b^		1.00	(Reference)	0.74	(0.55–1.01)	0.77	(0.56–1.05)	0.85	(0.62–1.17)	0.29
Female										
Cases, *n*	257	72		73		59		53		
Multivariate-adjusted ^b^		1.00	(Reference)	0.97	(0.70–1.35)	0.79	(0.56–1.13)	0.83	(0.58–1.20)	0.19
By smoking status										
Current or past smokers										
Cases, *n*	235	73		57		47		58		
Multivariate-adjusted ^b^		1.00	(Reference)	0.89	(0.63–1.26)	0.75	(0.52–1.10)	0.84	(0.58–1.21)	0.25
Never smokers										
Cases, *n*	285	89		69		73		54		
Multivariate-adjusted ^b^		1.00	(Reference)	0.76	(0.56–1.05)	0.85	(0.62–1.17)	0.78	(0.55–1.11)	0.23
By coffee consumption										
<1 cup/week										
Cases, *n*	173	99		36		21		17		
Multivariate-adjusted ^b^		1.00	(Reference)	0.72	(0.49–1.05)	0.66	(0.41–1.07)	0.80	(0.47–1.35)	0.11
≥1 cup/week										
Cases, *n*	403	81		107		105		110		
Multivariate-adjusted ^b^		1.00	(Reference)	0.88	(0.66–1.18)	0.78	(0.58–1.05)	0.81	(0.60–1.10)	0.15
By green tea consumption										
<1 cup/week										
Cases, *n*	44	29		9		4		2		
Multivariate-adjusted ^b^		1.00	(Reference)	0.98	(0.46–2.10)	0.69	(0.24–2.00)	0.43	(0.10–1.86)	0.23
≥1 cup/week										
Cases, *n*	532	151		134		122		125		
Multivariate-adjusted ^b^		1.00	(Reference)	0.84	(0.67–1.07)	0.79	(0.62–1.01)	0.86	(0.67–1.10)	0.18
By alcohol consumption										
<150 g/wk										
Cases, *n*	416	111		107		95		103		
Multivariate-adjusted ^b^		1.00	(Reference)	0.90	(0.69–1.18)	0.79	(0.60–1.04)	0.86	(0.65–1.14)	0.20
≥150 g/wk										
Cases, *n*	160	69		36		31		24		
Multivariate-adjusted ^b^		1.00	(Reference)	0.71	(0.47–1.06)	0.75	(0.49–1.15)	0.77	(0.48–1.25)	0.19
By BMI										
<25 kg/m^2^										
Cases, *n*	411	122		104		93		92		
Multivariate-adjusted ^b^		1.00	(Reference)	0.88	(0.67–1.14)	0.80	(0.61–1.05)	0.81	(0.61–1.08)	0.11
≥25 kg/m^2^										
Cases, *n*	141	46		33		31		31		
Multivariate-adjusted ^b^		1.00	(Reference)	0.78	(0.50–1.23)	0.80	(0.51–1.28)	0.93	(0.58–1.50)	0.71

Abbreviations: CI, confidence interval; HR, hazard ratio. ^a^ Age- and area-adjusted model adjusted for age (continuous), sex, and area (10 public health center areas). ^b^ Multivariable Cox proportional-hazards models were adjusted for area (10 public health centers area), age (years) and sex (men or women), smoking status (nonsmoker, past smoker, current smoker <20, 20–40, ≥40 cigarettes/day, or missing), history of diabetes mellitus (yes or no), family history of pancreatic cancer (yes or no), alcohol consumption (0, <150 g/week, ≥150 g/week, or missing), and body mass index (<25 kg/m^2^, ≥25 kg/m^2^, or missing).
